# Culture Circles in adolescent empowerment for the prevention of violence

**DOI:** 10.1080/02673843.2014.992028

**Published:** 2015-01-06

**Authors:** Estela Maria Leite Meirelles Monteiro, Waldemar Brandão Neto, Luciane Soares de Lima, Jael Maria de Aquino, Daniela Tavares Gontijo, Beatriz Oliveira Pereira

**Affiliations:** ^a^ Graduate Program in Child and Adolescent Health, Centre of Health Sciences (CSS), Universidade Federal de Pernambuco (UFPE), Av. Professor Morais Rego 1235, Cidade Universitária 50670-901, Recife, Pernambuco, Brazil; ^b^ Associated Graduate Program in Nursing, UPE/UEPB, Faculdade de Enfermagem Nossa Senhora das Graças (FENSG), Universidade de Pernambuco (UPE), Rua Arnóbio Marques 310, Santo Amaro 50100-130, Recife, Pernambuco, Brazil; ^c^ Institute of Education, Research Centre on Child Studies (CIEC), University of Minho, campus de Gualtar 4710-057, Braga, Portugal

**Keywords:** adolescents, high school, participatory action research, health education, violence prevention, youth empowerment

## Abstract

An action research based on Paulo Freire's Culture Circles was developed to implement a health education intervention involving adolescents, in collective knowledge construction about strategies for the prevention of violence. The data collection in the Culture Circles involved 11 adolescents and included observation and field diary, photographic records and recording. The educational action aroused a critical socio-political and cultural position in the adolescents towards the situations of vulnerability to violence, including the guarantee of human rights, justice and the combat of inequities; changes in the social relations, combat against discrimination and intolerance; expansion of access and reorientation of health services through intersectoral public policies. The intervention empowered the group of adolescents for the prevention of violence and permitted the inclusion of health professionals in the school context, from an interdisciplinary perspective, contributing to the establishment of social support and protection networks.

## Introduction

Adolescence represents a human development phase marked by intense organic, mental and behavioural transformations, which are subject to social, cultural and environmental influences. In this context, the adolescents are more vulnerable to different situations, such as violence for example.

Violence can be defined as follows:

the intentional use of physical force or power, threatened or actual, against oneself, another person, or against a group or community, that either results in or has a high likelihood of resulting in injury, death, psychological harm, maldevelopment, or deprivation. (Violence Prevention Alliance [VPA], 2014)

As a social phenomenon, violence influences the groups of adolescents and young people in different manners, compromising the rights to the protection of life and health, as well as social and family relationships. In Brazil, homicide represents the main cause of death in adolescents and young people between 15 and 24 years of age, particularly affecting young Afro-American males, living on the outskirts and in metropolitan regions of urban centres (Waiselfisz, 2013).

The complexity involved in violence in this group indicates some directions and factors of exacerbation in the communities: the relation between violence and drugs, poverty, social inequities, absence of investments in social policies, naturalisation and acceptance of the phenomenon, lack of community resources and changes in cultural values (Affonso et al., 2010; Reis et al., 2013; Silva et al., 2014). Devising prevention strategies is not an easy task, but there are promising routes. Specifically, the health sector has been attempting to establish intersectoral work, committed to the protection and health care of adolescents within an integrative perspective of individual and social priority rights.

The approach of the violence phenomenon, based on the biomedical model, limits the health professionals' activities, making prevention insufficient for the community demands, as this dominant model is marked by a logic of vertical relations (Oliveira, Almeida, & Morita, 2011), absence of listening and welcoming and sometimes blaming posture in the attempt to find the causes of the phenomenon. It is fundamental for the professional activities to be guided by humanistic and bioecological paradigms, which promote a holistic view of the individuals inserted in the different contexts.

Departing from this understanding is the National Policy for Comprehensive Adolescent and Youth Health Care in Brazil (Brazil, 2010a), whose great challenge is to programme, mobilise and develop actions that fully attend to the demands related to the distinct vulnerabilities in terms of adolescent health, particularly the establishment of a culture of peace. The fundamental strategic axis this policy rests on is the empowerment of the adolescent group as a protagonist in health promotion practices to prevent violence.

Intervention proposals to prevent violence have been experienced around the world, using strategies that operate in individual, social and community components. In view of the strong relations between violent practices and the historical–social context the violence is produced in, successful interventions are those that intend to focus on the empowerment processes that involve the unveiling of reality (awareness raising) and the transformation of social relations of dominance. Examples are the studies by Reischl et al. (2011), Berg, Coman, and Schensul (2009), Black et al. (2009) and Bosma, Komro, Perry, Veblen-Mortenson, and Farbakhsh (2005), which considered important mechanisms in the prevention of violence, young people's perspective on the actual context of their lives based on critical dialogue and juvenile training for the planning and implementation of prevention programmes.

As an essential health promotion strategy, empowerment is considered a development process of personal and social skills, with a view to gaining control and decision power in priority issues of life in society. Its use requires the adoption of teaching approaches based on liberating pedagogical trends, as proposed in Paulo Freire's Culture Circles (Brandão, 2005; Freire, 2008). Adopting this educational model in interventions with adolescents related to violence prevention implies privileging the subjects' experiences in the participatory processes, which grants the adolescents the power of speech, of featuring, of freedom of artistic expression, turning them into actors on the stage of health interventions. In addition, other factors motivated the researchers in this study, which were limited action of health professionals in the school sphere, lack of knowledge about the importance of active methods in the spaces available to discuss complex social problems, weaknesses in the school environment to cope with the violence problem and the need for studies in distinguished cultural contexts whose scope contains the education of adolescents as agents of change to deal with problems in the community.

The objective in this study was to implement Culture Circles as an educational invention in health involving adolescents, in collective knowledge construction about strategies to prevent violence.

## Conceptual base

This study was based on Paulo Freire's critical pedagogical theory as a theoretical and methodological resource to elaborate the educational intervention in health with the goal of preventing violence. Paulo Freire gained global repercussion when he proposed a new educational paradigm, based on concepts such as dialogue, autonomy, hope, relation oppressed/oppressor, humanisation and emancipation. Freire (2010) describes the need to reflect on the exercise of a conscious and critical educational practice as a form of intervention in the world, committed to the principle of democracy that rejects any form of discrimination, domination and integrates an attitude of innovation and renewal, in the possible that change is possible. In that perspective, the educator inaugurates the popular education movement through the development of the youth and adult alphabetisation method, idealised in the so-called Culture Circles, deriving from his experience in the Brazilian Northeast in the 1960s, a moment in history that was marked by political crises like the military coup.

The Culture Circles Freire proposed are considered dynamic spaces of learning and knowledge exchange that value the group experience and promote its participation in the construction of collective, contextualised knowledge that is committed to the transformation of reality. Organised in the form of circles, the individuals meet in an educational process that is aimed at investigating themes of interest to the group. The key elements guiding this process are dialogue and problematisation when the students are invited to confront the situations experienced in their daily life with a view to further critical feedback (Brandão, 2005; Freire, 2008, 2011).

Freire's thinking can be considered contemporary, and beyond an education method or theory, it demonstrates potential as a new worldview, perceiving the individuals as subjects capable of making history and culture. The praxis of educational action as a political act of freedom should promote autonomy in the students, aiming for liberation, not only in the cognitive, but essentially in the social and political fields, making them work with a view to a better quality of life for themselves and the community they belong to (Freire, 1997). In public health, Paulo Freire's theoretical and philosophical constructs have influenced the processes of empowerment education in health promotion (Souza et al., 2014; Wiggins, 2011). The connection established between health promotion and Freire's critical–social thinking have permitted the acknowledgement of intersubjective, behavioural and ethnic-cultural aspects as inherent in the production of health care, and the deepening of political debates on the supply of educational strategies that can transform the vulnerability situations the adolescents are exposed to.

## Method

**Figure 1:**
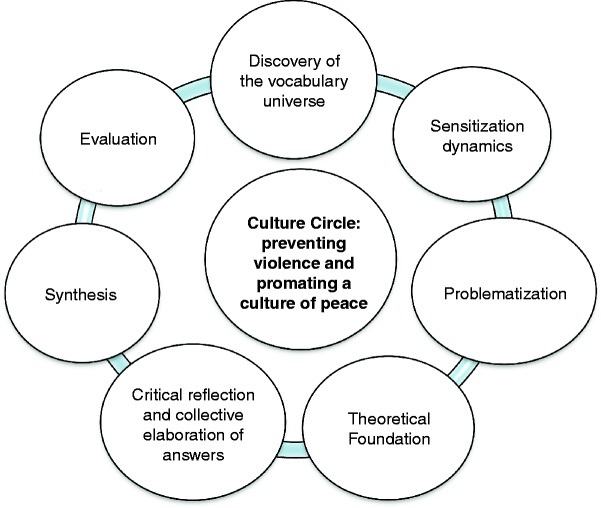
Phases of Paulo Freire's method proposed by Monteiro and Vieira (2008) and applied in this study.

Action research with a qualitative approach based on Freire's pedagogy of Culture Circles. Paulo Freire's method, as a theoretical and methodological resource in research, consists of three dialectic phases: *thematic investigation*, when the educators/entertainers discover, in the students' vocabulary universe, the generative words and thematic contents of real life; *theming*, when the themes are coded and decoded through the dialogue, the symbols gain social meaning, the participants gain awareness of the world experienced:and *problematisation,* a phase with a process of action–reflection–action, where the participants in the Culture Circle attempt to overcome the first magical view through a critical and social view, acknowledging the limits and possibilities to transform the context experienced (Freire, 2008, 2011). In this study, the health education model proposed by Monteiro and Vieira (2008) was used, adapted from Paulo Freire's Culture Circles, as displayed in Figure 1.

To develop this research by means of the Culture Circle, the researcher needs to pay attention to what is said. The statements, conversations, phrases, interviews, discussions, within or beyond the circle, everything is loaded with themes from the community, its topics, its life (Freire, 2011). Hence, the choice to develop a Culture Circle aims to trigger a participatory experience of knowledge construction, with emphasis on dialogue and experience exchange, which is capable of provoking individual and collective reflections with a view to gaining critical awareness and active postures towards the experience (Heidemann, & Almeida, 2011; Monteiro & Vieira, 2010).

The innovation proposed in the Culture Circles is characterised by the circularity and interrelation of its phases, resulting in a group that participates more in the dialogues and debates, and also generates autonomy and commitment for decision-making. The application of this method requires that the health professionals serve as ‘entertainers’, going against the role of teachers in the traditionalist view, which is that of holders of knowledge (Monteiro & Vieira, 2010). The role of the animator is to coordinate the debate, problematise the discussions for opinions and reports to emerge and which does not remain limited to teaching, but takes interest in learning with the group (Freire, 2008).

### Research scenario and participants

The research context was a public school from the state-owned network in the city of Recife, Pernambuco, in the Brazilian Northeast, located in what is considered as a vulnerable community, with high homicide rates and the presence of drug traffickers. The school is part of the State Program for Full-Time Education, with extending teaching in the afternoons and a lack of primary health care services.

The research proposal was agreed upon with the coordination of the State Health Department and later presented to the school board and teachers. To select the participants, the research was disseminated in all secondary-education classes taught during the morning period. The adolescents received a folder with the title and origins of the research, how to participate in the educational group and a registration form that asked the adolescents to express their interest in participating in the Culture Circle. The criteria to select the adolescents who formally expressed their intention to participate in the action research were: being enrolled in and regularly attending the pedagogical activities and having completed the registration form correctly.

The choice to work with a non-probabilistic intentional sampling is based on the proposal of the adolescents' free and spontaneous participation in the final composition of the sample. Strengthening the ‘spontaneous and conscious participation of young people is essential for the development of the Culture Circles, whose dialogic nature and awareness-raising constitute relevant frameworks of this method’ (Monteiro & Vieira, 2008, p. 71). Thus, 40 students registered but, in view of the dialogical and participatory nature of the adopted teaching approach, small groups were constituted, involving 11 adolescents from the first and/or second year of secondary education, in the age range between 15 and 19 years, including six female and five male participants.

### Data collection

The data collection took place at two moments: visits to the school made between September and October and an educational meeting guided by the principles of the Culture Circle, held in November 2011, including participant observation with notes in a field diary, photographic records, recordings and play techniques with the elaboration of drawings and the use of play dough. The researchers made five visits in the afternoon period, which took one hour each. The length of the Culture Circle was 2 hours and 30 minutes, in the school facilities, in the afternoon period, after the students' pedagogical activities. For the recording and photographic records, an undergraduate student participated who received preliminary directions as to her behaviour during the recording. The student was an academic culture and community service grantee who was active in health education activities at the school, enhancing the students' acceptance. It should be highlighted that the inclusion of the grantee allowed the researcher to focus on the observation and coordination of the debates and play activities.

The use of the play dough and free drawing as projective techniques for the data collection granted access to latent and hidden contents in the adolescent imaginary, which are not manifested directly. In combination with the subjects' discourse, this permitted a consistent, in-depth and rich analysis (Teixeira, Paiva, Nóbrega, & Nitschke, 2013) in view of the complexity and plurality of the theme violence.

### Data analysis

To organise and treat the data, data triangulation analysis was adopted, as this permits the observation of the investigated reality from distinct angles, allowing the researchers to get deeper into the reality and furthering the validity and reliability in qualitative research (Denzin, 2009; Flick, 2014). Thus, to systemise the data collection, the researchers described all events that happened in the Culture Circle in detail. The information in the field diary was narrated, registering the contextual information and discussions and reflections evidenced during the meeting, with voice recordings, photographic records and the transcription of the material produced in the recording. Thus, the meaning of the play experience for the actors involved was apprehended and the study participants' aplomb could be accompanied. The compilation of this material led to the design of a single, homogeneous and representative transcription (constitution of the research corpus), presented in the order of the phases of the Culture Circle. Finally, the material was discussed and analysed in the light of the relevant literature on the theme, in a critical-dialectic movement.

### Ethical research procedures

Approval for the study was obtained from the Research Ethics Committee at Universidade de Pernambuco, under Protocol 076/11 and registration CAEE (stands for Certificado de Apresentação para Apreciação Ética) 0062.0.097.000-11. In compliance with Resolution 466/2012 (Brazil, 2012) on research involving human beings, the parents or responsible caregivers and the adolescents assented through the signing of the Free and Informed Consent Form. The adolescents' privacy was guaranteed through the use of pseudonyms, followed by gender and age identification. The adolescents chose the pseudonyms because they represented personal characteristics, during a dynamic, casual introductory activity.

## Results

### Visits to the school – survey of the vocabulary universe

The discovery phase of the adolescents' vocabulary universe included an activity started before the Culture Circle, through individual preliminary contact with the participants on the aspects that permeated the violence problem, based on which the generative words could be extracted that were to be addressed during the meeting. In addition, other views on the planning were raised, which had not been concluded and was therefore flexible.

All of the adolescent live in the community near the school and mention daily contact with situations of violence. They consider the school as an important place for their life and have positive expectations towards the future. As regards these adolescents' sociodemographic characteristics, it was identified that, in their family structure, the mother figure is the most present and responsible for sustaining the children, while the father is a distant member, whether due to separation and the establishment of a new family or because of a single mother. Concerning the types of employment, the majority were low-education professions, such as domestic servant, sales clerk, drugstore attendant, school cooks and chambermaids.

As perceived, the adolescents have countless artistic skills, including painting/drawing, modelling, recycling, dancing, singing and poetry writing, besides the valuation of the regional culture, with activities such as capoeira, maracatu and frevo. This potential is neither identified nor valued in the traditional teaching model though.

Apprehending the adolescents' social sphere, getting to know their likes, preferences, habits, histories and life experiences, as well as the way they relate to others, was very important in this phase and permitted showing the school community's difficulties to cope with the violence problem. Located in a community with high rates of violence and drug consumption, the school cannot escape from the surrounding problems.

### Educational intervention with Culture Circle – preventing violence and promoting a culture of peace

For the *sensitisation activity*, an undergraduate nursing student with vocal and musical skills participated. The Culture Circle activities started at the sound of a guitar with a very happy, sensitive and involving repertoire, playing romantic, rock and Brazilian Popular Music. The adolescents themselves requested these styles.

The intention of the activities was to show the participants' engagement, through gestures, expressions and subtle laughing, who were singing aloud, attracted by the power of the music, demonstrating how important it is to permit moments of reflection, learning jointly and valuing other people with their history and culture, with a view to collective action.

To proceed with the Culture Circle activities, the *problematisation activity* started, when the following question was raised in the group: How can violence be prevented and a culture of peace promoted? Therefore, the participants were divided in two teams. A fictitious situation was proposed in which each team consisted of different professionals, hired by a public organisation to jointly elaborate a social project that was aimed at preventing violence. The teams were stimulated to display posters with drawings and images that answered the question raised. For this playful activity, didactical–pedagogical material was made available, such as colour pencils, crayons, colour glue, markers, play dough, cardboard, legal-sized paper, pencils and a rubber.

Thus, the first adolescent team elaborated a poster with the individual drawings (Photograph 1):

**Photograph 1:**
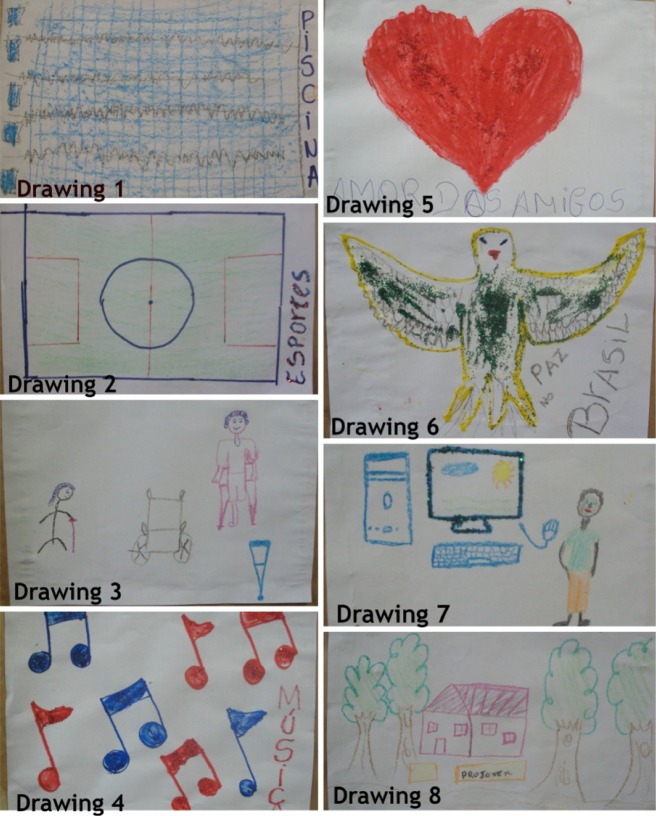
Poster on the strategies to prevent violence and promote a culture of peace, elaborated in the first adolescent team.

**Table:** 

Individual construction of strategies to prevent violence and promote a culture of peace	
(Creativity, M, 18 years) Drawings 1 and 2	…all young people deserve to be happy at least once in life, without any distinction of color or race, where they can go to college, everyone needs opportunities… If I were an important person, I'd make leisure areas for children to play, I'd invest in sports areas like swimming, soccer courts. This can help to get the children and youth off the streets…
(Joy, F, 17 years) Drawing 3	The people who are disabled, many keep on laughing and calling them crippled, I find that a form of violence… and there are many places that don't even have quality to receive physically disabled people or elderly, the streets have no organization for these people… many people do not respect the rights of the elderly… we also need to consider these things as situations of violence…
(Overcoming, F, 19 years) Drawings 4 and 7	…also offer further opportunities, such as: access to informatics, to new things, investing in what's good in us, in music… engaging people also offers great personal help and for the professional career…
(Studious, M, 16 years) Drawing 5	Exclusion is a kind of violence and we need to do the opposite – inclusion –, bring people who are excluded, such as the physically disabled, elderly, gays, black, street dwellers, people with different religions for life in society…
(Love, F, 18 years) Drawing 6	We wanted to show that all this is a way to help, to promote peace and minimize the violence, because we think of all this as a form of rescuing, valuing the people who feel excluded from society nowadays…
(Friendship, M, 15 years) Drawing 8	It is important to create non-governmental centers or organizations that rescue young drug addicts so that they go back to school

The second adolescent team presented a poster jointly elaborated with play dough (Photograph 2):

**Photograph 2:**
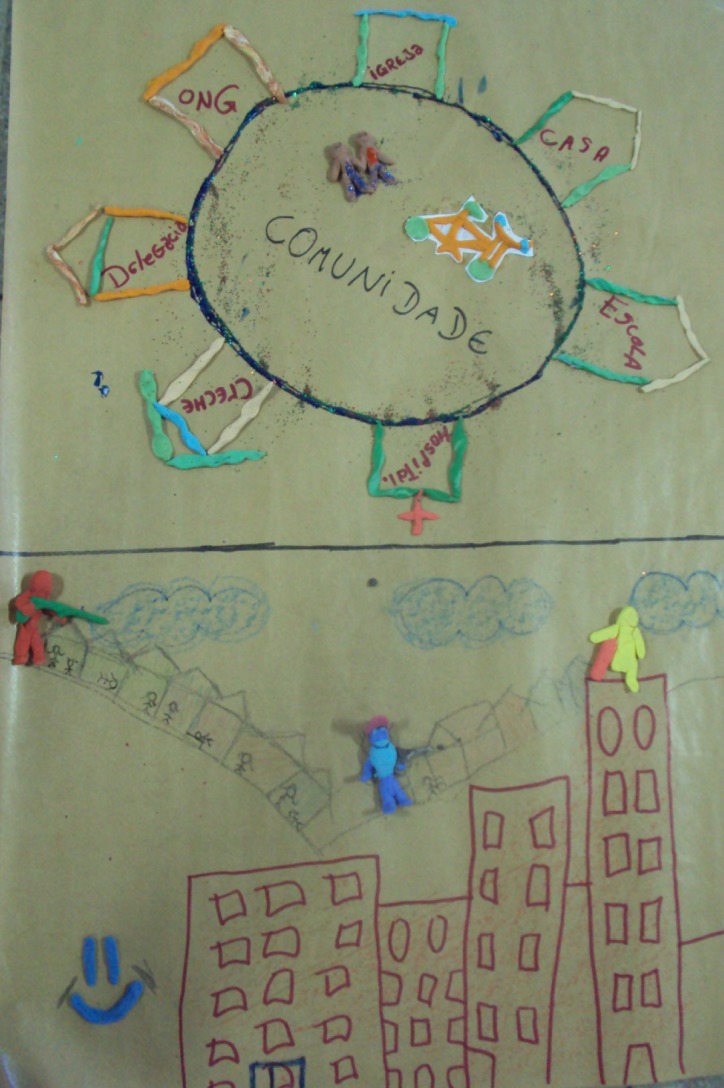
Poster on the strategies to prevent violence and promote a culture of peace, elaborated in the second adolescent team.

**Table:** 

Collective construction of strategies to prevent violence and promote a culture of peace	
(Life, F, 16 years)	The community here is full of hills, with some surround buildings representing rich people and poor people with social differences… in the community right behind, shootings are common, adolescents using guns and drugs, innocent people being hit by lost bullets… and in these buildings nearby, there are people with money, who are luxury and know the difficulty in the slums, but don't care and are living their life happily
(Hope, F, 17 years)	It's as if there were two worlds, right! Where there's this perfect world where nothing ever happens, where only money matters and the leisure and what happens out there does not matter… I think peace is a right of all…
(Courage, M, 15 years)	But, if the world lived in union, which we represented through this circle where we need each person… then we thought of union among the schools, hospitals, kindergartens, community precincts, social projects of non-governmental organizations, churches and our relatives too
(Dream, M, 16 years)	The school should know our family better, visit our homes, know our problems… and the school should also work together with the hospitals and health service, because often there are situations in which we need…
(Perfectionist, F, 15 years)	These links we are showing together with good communication could perhaps make the difference and help to reduce the violence, due to the fact that we're all in the same fight, together… the Circle drawn was considered because it represents an alliance, and an alliance does not break, it is endless…

The *reading activity* served to appreciate the text: ‘Promotion of the Culture of peace’, taken from the manual of the Brazilian Health Department (Brazil, 2010b), entitled: Care guidelines for comprehensive care to child, adolescent and family health in situations of violence: orientation for managers and health professionals.

In the selection of the texts needed to provide solid theoretical foundations and which would contribute to the discussions and reflections raised in the group, texts that were neither very long nor tiresome were needed, so as not to discourage the adolescents' reading, which is that important in the critical reflection process.

During this phase, Joy's statement revealed that the work in the Culture Circle is interrelated with the adolescents' life context, as shown:

According to me, each of these houses modeled would be a way to contribute to combat the violence, generating more peace… starting with the family, then the school, church…. (Joy, F, 17 years)

In this perspective, it was clear that a cyclical, continuing and live process triggers the educational action resulting from Paulo Freire's Culture Circle, in the students' historical, social and cultural context, raising challenges for the group and leading them to critical awareness through debates.

With a view to effectively contributing to the experience of an intense communication process, but with specific intentions to enhance the group participants' empowerment and autonomy, finally, the *synthesis activity* was undertaken to identify the meanings and data that were considered most noteworthy in the educational action. Thus, the adolescents' considerations emphasised the need to bring down barriers that produce differences in society, as well as to enhance the knowledge repertoire about the social reality the violence is established in, including the commitment to intervene in it.

Any Culture Circle has an assessment as its final phase, which is not mediated by classifications and grades, but by the participants' self-assessment of their experience in the teaching-learning process, focusing not only on the subjects, but also on the researcher/entertainer's activities in the Culture Circles (Monteiro, Cavalcanti, Aquino, Silva, & Lima, 2013). For this *assessment activity*, an open space was stimulated to make the adolescents feel at ease to discuss the experience and anything else they found interesting, while collectively constructing the knowledge. The following statements emerged:

Thinking as if we were developing a social project for ourselves, the adolescents, was really cool, as we discovered our own and the community's strength…. (Creativity, M, 18 years)

It stimulated our imagination, promoted joined learning…. (Love, F, 18 years)

## Discussion

The educational action offered a favourable environment for the adolescents to express themselves easily and naturally, contributing to the critical reflection proposed in the Culture Circle (Monteiro & Vieira, 2010) and mobilising them for the construction/deconstruction/reconstruction process of reality, permitting the production of more conscious postures towards their social role.

The problematisation activity gave rise to dialogue, knowledge exchange, emphasising the valuation of popular knowledge, experiences and particular ways of dealing with the questions that involve the daily human life with its bio-psycho-sociocultural relations. Thus, the adolescents engaged in the coding and decoding process of the extreme circumstances involved in the violence problems and their countless sides and forms in the life of each individual. This teaching-learning process through the apprehension of reality strengthened the critical reflection and commitment to contribute with important elements to consider the prevention and combat of violence.

In the appreciation of the adolescents' playful construction in the first group, Joy's testimony showed the perception of violence involving disabled and elderly people. These individuals are considered socially excluded because they are victims of violence. Their physical condition is described with the use of derogatory words and an urban space is lacking to guarantee access to social resources and provisions. Besides, legally established determinations are not complied with.

Minayo (2005) and Silva et al. (2014) consider the social exclusion of certain groups as a form of structural violence, including social inequalities, violation of human rights, manifestation of poverty, of precarious conditions in the community and of discrimination. In that sense, Studious' statement revealed concerns with the promotion of social inclusion by recovering the adolescents and youth's talents, skills and creative capacity in the creation and recreation of different realities.

Other statements highlighted the creation of rehabilitation centres for adolescent and young drug users, as well as investments in leisure, sports and culture areas, professionalisation opportunities, access to education, valuation of young talents through music and other artistic activities. These aspects, for example, are hardly explored in schools' political-pedagogical projects, which have great potential to develop sports activities and recreation options.

In the same direction, Peres, Bodstein, Ramos and Marcondes (2005) add that leisure, sports and culture activities in poor areas of cities join moral and ethical values that can modify children and young people's perception of ‘life’, distancing them from violence and the crime world, thus constituting a resource to enhance the possibilities of changes in reality. Therefore, the articulation between public sports and leisure possibilities, urban policies and policies that combat violence needs to be considered, with a view to promoting a better quality of life for the community.

In addition, the adolescents from the first team raised the theme of the peace culture, underlining that to construct it, attitudes of respect for human rights are needed, with a view to guaranteeing plural spaces where differences can live together free from violence (Beck, 2012; Grossi, Aguinsky, Brancher, Oliveira, & Schneider, 2005).

It was identified that the two themes of adolescents converged on the central themes addressed in the problematisation phase. Despite the complementariness in the contents, in the second theme, a proposal to overcome the violence could be identified, moving towards the ideological thinking that sustains the expanded concept of health.

The adolescents in the second team pictured a common reality in large Brazilian urban centres, demonstrating situations of economic, social and spatial segregation, which impose a form of cultural organisation that is peculiar to families living on the outskirts, contributing to situations of vulnerability to violence, such as criminality, exclusion, stigmatisation and discrimination. Living in these vulnerable communities sometimes makes one think of actual social confinement in tenements and precarious houses, where their problems are isolated from the surround world. They are not only excluded from access to goods and services, but also from enjoying the city itself.

The representations of violence, in the family as well as in the community, were evidenced in the research by Reis et al. (2013) and Fowler and Braciszewski (2009) as a phenomenon that is almost always associated with drugs use, poverty, precarious infrastructure, such as deficient garbage collection and architectonic barriers in the large cities, difficulties to get transportation and access to health. According to the adolescents, these symbolic manifestations of violence are linked to the social, cultural and economic levels experienced in the community.

In parallel with this reality, in a circle, the adolescents represented small houses to allude to social sectors that intend to cope with the situations of violence experienced, based on the acknowledged need to structure a support network with schools, hospitals, kindergartens, community precinct, social projects of non-governmental organisations, churches and families. The adolescents do not only indicate a vulnerable reality, but also think of transforming it. They are concerned with identifying the resources and equipment in the community that can enhance changes.

The construction of this collective knowledge reveals the meaning of the beliefs in the participating group's potentials and creativity, in an educational education based on Freire's principles. In view of their understanding of the complexity of the theme violence, the contents the adolescents produced led to the construction of a daring proposal, involving principles such as an intersectoral approach, integrality, accessibility, referral and counter-referral, citizenship, participation, mobilisation and, finally, personal and social empowerment. These principles constitute the solid base of the Brazilian Unified Health System, in which issues of violence have been a source of intense debates and discussions in public policies.

Perfectionist and Courage's reports expressed that investments in the constitution of networks, including families and communities, besides public and non-governmental organisations, could create prevention and coping strategies to respond to the challenges life in situations of violence demands.

Intersectoral and complex actions, in networks, pushing back frontiers and responding to the demands through exchanges in health practices also mean attracting different social groups to the debate and, together, building collective projects that move towards a fairer society (Meirelles & Erdmann, 2006). Nevertheless, despite the needs and potentials of networking, the processes leading towards integrated and interdisciplinary actions remain incipient (Eyng, 2013).

It should be highlighted that the idea of the networking strategy to safeguard rights is included in article 86 of the Brazilian Statute of the Child and Adolescent, which determines: ‘The policy of compliance with the rights of children and adolescents will be put in practice through an articulated set of governmental and non-governmental actions, involving the Union, States, Federal District and the Cities’ (Brazil, 1990).

In their testimonies, Dream and Courage reveal concerns with including the family in the alliances for the constitution of support and social protection networks, and also recognises the strategic role of the school in that sense. When the school interlinks with the surrounding world, possibilities are created to break frontiers and narrow knowledge, values and cultures through actions that can start with (and in) the school. Nevertheless, both families and schools face many difficulties to become partners in networking.

The understanding of the family's role among education and health professionals and tutelary counselors is a source of concern, as it seems that the family's view remains more closely linked to the idea of a space of violation than protection and guarantee of rights. Hence, few of them consider the family as a partner in networking. (Eyng, 2013, p. 258)

Dream's statement also emphasised the importance of the relation between schools and health services, with a view to an easier understanding of the adolescents and families' health needs. In that perspective, the presence of the nurse(s) and other health professionals at the school gains relevance, strengthening the commitment to health promotion by stimulating co-participation, promoting discussions, encouraging technical debates and presenting their perspectives on the processes of health, disease and care, besides approaching the social relations between education and health professionals (Rasche & Santos, 2013).

Next in the Culture Circles, while reading the material, the adolescents highlighted some of its parts in pencil. At the end, they mentioned that in the playful activity, the teams addressed situations the text was identifying. In addition, the adolescents underlined the need for changes in human social relations, with a view to reducing the violence and promoting peace culture with social justice, equality, fighting poverty, prejudice and discrimination, respect for minorities, education for all and freedom of expression.

The opportunity to have students, teachers, adolescents, citizens, professionals from different areas, in short, a wide range of social actors sit in a circle, talk and reflect, sharing the same objective and willing to act, offers the possibility to start on a trajectory that can overcome violence (Melo et al., 2007).

Based on the combination of popular and scientific knowledge, which the reading provided, it was perceived that the adolescents were able to revisit and problematise their reality, using the power of knowledge to critically consider that reality. Departing from that understanding, Freire (2010) considers that this attitude makes it easier for individuals to take a political position, stimulating their critical awareness that permits a more global understanding of the social phenomena and the perception of new possibilities for social construction, of the ‘non-experienced possibility’ in the positive transformation of reality.

In the synthesis activity, the adolescents discussed the importance of acknowledging their social role. Thus, despite sometimes contributing to attitudes of violence, humans can divest themselves of these practices and start to construct a new worldview in which our ‘weapon’ against violence is:

Education for peace, solidarity, respect, love and compassion, so as to relight the flame of the human values of living and living together, in defense of human dignity, respect for life, for the planet and the valuation of cultures. (Monteiro, Rolim, Machado, & Moreira, 2005, p. 343)

In the assessment of the Culture Circle, the adolescents considered that the possibility to construct knowledge based on the promotion of spaces of interaction, engagement, self-esteem, joy and responsibility permitted the group's evolution towards the elaboration of a proposal to cope with the situations of violence, despite the group members' distinct worldview. They dialogued, launched thoughts and approached their understanding to achieve a common goal.

In addition, the Culture Circle offered the adolescents a moment of discovery and renewal, believing in their potentials and even more in the potentials of their social context, where despite the adversities, opportunities are present to provide resources for social transformations. Therefore, the expressions and creations of the adolescents which emerged from the playful and the symbolic should be valued and motivated, which is often denied in formal education.

Working in a perspective of social transformation means attracting the adolescents to the debate on relevant issues in their life, aiming to look for solutions based on small individual problems manifested to the group. When a micro-problem reveals to be common to all, the adolescents perceive the group's strength as a means to transform their reality, leading towards a political action, in the construction of a fairer, more equal, participatory and responsible society (Cardoso & Cocco, 2003).

### Final considerations

In this study, an educational intervention was implemented with adolescents, addressing the construction of proposals to prevent violence. Working with such a complex theme demands a sensitive, welcoming, dynamic and innovative attitude in the construction and deconstruction process of thoughts and attitudes. In this process, Paulo Freire's Culture Circle emerged as a health education strategy, enhancing reflection-action as a result of collective construction, through the creation of emancipatory spaces for the strengthening of the community.

The school context was proper as fertile ground for the development of dialogical, critical and reflexive educational practices, in which the youth was valued in a protagonist role, given the importance of this environment in the education of children and adolescents for citizenship. This permitted approaching the daily life of adolescents, transforming their perception of reality in the school, family and community sphere and driving the construction of collective action plans, which consider the determining and conditioning factors of health in the production and reproduction of violence.

The adolescents' effort was verified to participate in the discussions and debates promoted in the Culture Circles, culminating in the strengthening of the interaction between the entertainer and the participants and the exaltation of feelings like high self-esteem, freedom to express oneself, desire to change, study, win and dream, breaking the prejudice and social stigma of adolescents living in poor communities.

The dialogue produced in the Culture Circle furthered the adolescents' inclusion in the teaching-learning process and encouraged them towards the collective elaboration of strategies aimed at contributing to the prevention of violence. The constructed proposals were based on protective, social, cultural and political elements, which were expansion of intersectoral public policies, reduction of inequalities, social inclusion, greater opportunities for the adolescents and youth through investments in leisure, sports, cultural and artistic activities for the exercise of citizenship, greater access to health services, expansion of organisations that develop non-governmental actions and the family's role in the constitution of support and social protection networks.

In this process, the work with the play dough and the use of free drawing were projective techniques capable of linking the reality to the adolescents' imaginary, revealing contents that enhance the coping with contexts of vulnerabilities and violence. In addition, their use demanded that the researchers worked on sensitivity together with the actors involved, with a view to the acceptance of the playful proposal, so as to add a wealth of meanings in the apprehension of the empirical material.

The importance of intervention programmes aimed at unveiling and preventing situations of violence should be emphasised due to its impact on children and adolescents. More than physical marks, it causes psychological, cognitive, emotional and spiritual losses. In addition, the school alone is unable to deal with the social burden of burden, leading to the invisibility and denial of the phenomenon, which the adolescents experience in their daily life as routine practice.

The adolescents' aplomb in the Culture Circles highlights the importance of young people serving as protagonists in the discussions and deliberations on public policies of interest to this age group. Promoting the young people's participation in a democratic and solidary pedagogical process is fundamental for the strengthening of the youth's leading role as an action tool that triggers positive changes in the political-social reality. Thus, the adolescents' activities are envisaged as true ‘managers’ in the institutional spaces so that the social policies are finally transformed into practical strategies capable of dealing with the dilemmas and conflicts of the contemporary society.

### Implications for practice and research

The development of this study reaffirms the principles adopted in the current initiatives to prevent violence, originating in the Brazilian Education and Health Departments, such as the School Health Program, in Decree 6.286, issued in December 2007, whose challenge is to articulate the health and education contexts in defence of the rights and protection of adolescence and youth, and to strengthen the commitment to these individuals' health promotion and the exercise of citizenship.

The study results suggest that violence prevention programmes can support adolescents and young people to gain social competences that turn them into agents of change for the problems experienced in the community. In addition, when considering prevention, working in an intersectoral and comprehensive perspective is fundamental for the strengthening of social support networks, which demands combined action between the management and other public and non-governmental spheres and the community, as well as interdisciplinary activities that involve different professionals. Within a social macrostructure, such strategies can contribute to the definition and implementation of public policies aimed at stimulating healthy environments of social life and the establishment of a culture of peace.

Finally, the Culture Circle served as a technology for community health care, as it favoured conditions for a dialogical, creative and systemised educational practice, thus promoting the empowerment of the professionals involved and of the adolescents. Its application in the health area involves opportunities and challenges. Opportunities to permit new methodological alternatives in the search for knowledge, and challenges to permit new faces for the qualification of health care committed to autonomy and social well-being.

### Study limitations

This qualitative intervention involved the local development of a critical educational action with school-age adolescents, with a view to collective knowledge construction on the prevention of violence. Due to the small dimension of the sample, the short length of the intervention and the inclusion of a particular group of adolescents through intentional sampling, the results cannot be generalised to the population. In addition, the study involving young people from vulnerable communities, with limited access to educational resources and public policy actions and relatives with low education levels and purchasing power. In view of Brazil's continental dimensions, with a multi-ethnic and multiracial population and the strong presence of social inequalities, the question remains whether other adolescent groups share the same opinions and have the same experiences.

Therefore, other interventions could invest in broader participatory educational approaches, also including a group of teachers, school board members and family members in the prevention of violence.
